# Book Review: Contribution à un Lexique Commenté en Science de l'Action Motrice

**DOI:** 10.3389/fpsyg.2020.609193

**Published:** 2020-11-10

**Authors:** Zhaïra Ben Chaâbane

**Affiliations:** Department of Educational Sciences, Higher Institute of Sport and Physical Education, La Manouba University, Tunis, Tunisia

**Keywords:** motor praxeology, traditional sporting game, internal logic, motor conduct, universals, ethnomotricity

The first edition of this book (1981) was entitled “*Contribution à un Lexique commenté en Science de l'Action Motrice,”* (Contribution to a commented lexicon in Science of Motor Action). Eighteen years later, a new and augmented edition (1999) is intitled “*Jeux, Sports et Société,”* (Games, Sports, and Society), with a subtitle “*Lexique de Praxéologie Motrice,”* (Lexicon of Motor Praxeology). The cover of the first edition was stark. The second one draws the attention with a sixteen century print showing players in action. The first edition is a testimony of the birth of a new point of view on motor actions. The second highlights one of the consequence of this new point of view: the interest of traditional games. Motor praxeology was bearing itself the highlighting of their richness.

Through the definition of the concepts necessary for an in-depth reading of motor situations, Parlebas proposes a scientific language specific to the field of physical activities and sports (PAS) and an innovative analysis of physical and playful activities. He founds “the motor action science,” which defines motor situation as a communication range. This means the abandonment of classic descriptive perspectives for the benefit of a conception mostly dynamic and relational, through a problematic linked to action. Of this “motor praxeology,” the author keeps the personalist aspect (Ullman, 1985): human behavior always has a meaning and motor skills cannot be confused with movement on its mechanical way. It is the “motor conduct” of a subject (Parlebas, 1981, 1999). There is no praxic activity without reading, without interpretation of the multiple informational elements of the situation, without relationship to the world and others. The subject is an acting subject, a subject in situation, a subject deciding the strategy and the action to be constructed.

We understand thenceforth the choice made in favor of traditional sporting games (TSG), widely represented in the book. For a long time condemned by the authorities, abandoned for the most disadvantaged, and neglected by research, TSG reveal their complexity. “Few authors have studied games on the field, in their operation, and in their original characteristics. The risk is strong to only present a game alchemy, with missing a rigorous study of true playful chemistry” (Parlebas, 2003, p. 2).

The modeling of structures of motor situations done by Parlebas in his lexicon reveals the extraordinary richness of TSG. Above all, he emphasizes the necessity to take into account the internal logic (Parlebas, 1981, 1999), an intrinsic reality of sporting games which expose the motor conducts. These conducts manifest according to the relationship they create between the actor and its environment: relationship with space, objects, time, and other actors. The analysis of this interaction, primordial step and essential for an objective understanding of the game, leads to a reconsideration of TSG: the inclusion of “affectivity, the key to motor conducts” (Parlebas, 1970, 2017), symbolic aspects, representations, communication, motor decisions, informational data, signs and codes, particularly abundant in TSG, induce on the field important changes in pedagogical interventions modalities, and a new way of conceiving research in PAS field.

The classificatory and modeling approach offered by the author, notably borrowed to theory of games and theory of graphs, comes to complete the identification of game: According to Parlebas, the system of interactions defined by the rules of ludomotor contract creates an organization of motor actions, whose configurations reveal basic structures of the game process. “*It's the body of rules that brings into play the rules of bod*y” (Parlebas, 2003, p. 3) as he said. These configurations are operational models, called “universals” of sporting games; therefore, with analysis of the network of TSG motor communications, of the structure of their score interactions, of the network of sociomotor roles, or of their scoring system, we can discover original models with complex functions, in comparison to institutional sporting games (Sport) (Parlebas, 1981, 1999), which turns out to be stiff and structurally uniform.

These are the distinguishing features, specific to the internal logic of sporting game, defined, analyzed and illustrated in the lexicon, which allowed to establish an objective comparison between the different PAS. They constitute the “identity card” of the game and notably rely on the properties of the game system itself, opposed to “external logic” elements, which characterize the context (public, stakes, players characteristics,…) (Parlebas, 1981, 1999).

The author makes us discover this way that, on the basis of a rigorous analysis of ludomotor structures, extrinsic elements of the game bring further clarifications and enrich the understanding of TSG, from the angle of their relationship to culture and the social environment in which they developed. Every motricity is an “ethno-motricity” (Parlebas, 1981, 1999). The frivolousness of TSG is only an appearance: they are in reality the mirror of the community to which they belong, and participate to the cultural identity of each society which represents original playful patterns, linked to their lifestyles (Lagardera and Lavega, 2003; Parlebas, 2003).

Parlebas' lexicon, directed toward the construction of scientific specific field, covers all motor practices as a whole and bring a fundamental contribution to the knowledge of PAS. It is an essential tool for anyone interested in motricity field, a compulsory reference in sporting games theory. This theory represents a control on the clearest set of situations in which we can study social action and the set of interactions.

The question of terminology, the main element of the lexicon, must be understood in a context of a new relevancy, the one of motor action. It refers to the construction of a new object, without giving up the contributions of various scientific domains.

The permanent dialectic “system/acting subject,” ubiquitous in the lexicon, turns out to be full of possibilities and allow the revelation of practices that were depreciated for a long time, which constitutes a real revolution.

## Author Contributions

The author confirms being the sole contributor of this work and has approved it for publication.

## Conflict of Interest

The author declares that the research was conducted in the absence of any commercial or financial relationships that could be construed as a potential conflict of interest.
